# Three-dimensional facial analysis of Chinese children with repaired unilateral cleft lip and palate

**DOI:** 10.1038/srep31335

**Published:** 2016-08-10

**Authors:** Siti Adibah Othman, Noor Airin Aidil Koay

**Affiliations:** 1 Department of Paediatric Dentistry and Orthodontics, Faculty of Dentistry, University of Malaya, Kuala Lumpur, Malaysia; 2 Centre of Paediatric Dentistry & Orthodontics Studies, Faculty of Dentistry, Universiti Teknologi MARA (UiTM), Shah Alam, Selangor, Malaysia

## Abstract

We analyzed the facial features of Chinese children with repaired unilateral cleft lip and palate (UCLP) and compared them with a normal control group using a three-dimensional (3D) stereophotogrammetry camera. This cross-sectional study examined 3D measurements of the facial surfaces of 20 Chinese children with repaired UCLP and 40 unaffected Chinese children aged 7 to 12 years old, which were captured using the VECTRA 3D five-pod photosystem and analyzed using Mirror software. Twenty-five variables and two ratios were compared between both groups using independent *t*-test. Intra- and inter-observer reliability was determined using ten randomly selected images and analyzed using intra-class correlation coefficient test (ICC). The level of significance was set at *p* < 0.0018. Intra- and inter-observers’ reliability was considered fair to excellent with an ICC value ranging from 0.54 to 0.99. Statistically significant differences (*p* < 0.0018) were found mainly in the nasolabial region. The cleft group exhibited wider alar base root width, flattened nose and broader nostril floor width on the cleft side. They tended to have shorter upper lip length and thinner upper vermillion thickness. Faces of Chinese children with repaired UCLP displayed meaningful differences when compared to the normal group especially in the nasolabial regions.

Surgical repair of cleft lip and palate (CLP) is usually carried out early in life, with cheilorhinoplasty performed at three months of age and palatoplasty accomplished between ages of six months to one year^1^. Regardless of the timing of surgery and techniques used, residual deformity and asymmetry around the nasolabial region seems to characterise the facial appearance of CLP patients^2^. Among the secondary deformities observed after corrective surgery of CLP are wide alar implantations, short and flat upper lip, hypoplastic maxilla leading to upper lip retrusion and flat nose^3^. These abnormalities have an important influence on facial attractiveness and psychosocial well-being. CLP patients are more shy and socially inhibited when compared with non-cleft individuals. They also reported being teased in their childhood and adolescence; and are often stigmatised in social situations^4^. Comprehensive assessment of a multitude of aspects of CLP is essential. Whilst patient satisfaction^5^, psychosocial well-being^6^, speech^7^, and dental arch relationship^8^,^9^ have been thoroughly assessed, evaluation of facial appearance is limited.

There is diversity in the assessment of cleft-related deformity and this can be broadly classified into quantitative and qualitative assessment. Qualitative evaluation of nasolabial appearance involves subjective perception of deformity without performing any measurement on the stimulus material, which can range from coloured photographs, projected colour transparencies to on-screen digital photographs^10^. Asher-McDade *et al.*^11^ developed a standardised method to assess nasolabial appearance of patients with unilateral cleft lip and palate (UCLP) using standard coloured frontal and lateral photographs. The nasolabial area is rated using a five-point ordinal scale, 1 being very good appearance and 5 being very poor. Although this method is simple and quick, it is also rather subjective and relies heavily on judges’ experience and familiarity with the technique.

Quantitative analysis of cleft-related deformity involves facial measurements between specified facial landmarks. The anthropometric evaluation can be conducted directly on live subjects^12^ or indirectly on plaster casts^13^, two-dimensional (2D) photographs^14^, and three-dimensional (3D) imaging^15^,^16^,^17^,^18^,^19^,^20^,^21^,^22^,^23^,^24^,^25^,^26^,^27^. While direct subject measurement was thought to be the gold standard, it has many disadvantages which include the fact that it is time consuming, requires significant patient cooperation and poses risks of injury especially when involving measurement around the eyes. 3D imaging, for example stereophotogrammetry and laser surface scanning, have been validated and proved to be of equal standard to direct measurement. The values recorded by 3D systems appeared to be accurate and reliable for clinical use^28^,^29^,^30^,^31^,^32^. A recent systematic review was conducted to assess the treatment outcome in patients with cleft lip and palate using 3D technologies^33^. Soft tissue analyses were performed with either stereophotogrammetry or laser surface scanning as these techniques seem to be reliable methods for quantitatively measuring asymmetry and 3D changes in soft tissues after treatment. Another systematic review implied stereophotogrammetry imaging systems are more reliable and may have become the new gold standard^34^.

There has been increasing number of literature on anthropometric measurement of craniofacial complex pertaining to CLP. However, in Malaysia, data on 3D facial analysis for repaired UCLP is only available for Malay children aged 8 to 10 years old^20^ and adult patients aged between 18 to 25 years old of Malay ethnicity as well^25^. Even though the incidence of CLP in Malaysia is highest among Chinese, currently there is no study documenting facial measurements of Chinese children with repaired UCLP using 3D technology. Therefore, the aim of this study is to investigate the facial measurements of Chinese children in Malaysia with repaired UCLP and compare the results with a control group using 3D stereophotogrammetry imaging system. The specific objectives are to determine the facial measurements of Chinese children with repaired UCLP and those in control group and to compare the facial measurements between the cleft and control group. Our research hypothesis was that there is significant difference in the facial measurement between the cleft and the control group.

## Results

Recruitment of subjects took place from January 2013 to November 2014 and the final study sample consisted of 60 children aged 7 to 12 years old. Twenty (12 males and 8 females) were in the UCLP group with mean age of 10.22 years old and 1.81 standard deviation. Meanwhile the control group consisted of 40 samples (25 males and 15 females) with mean age of 10.03 years old and 1.51 standard deviation. There were no any significant sexual differences (*p* = 0.851) in both cleft and normal groups, hence the analysis of the male and female samples in both groups were combined. The data were examined for normality of distribution using the Shapiro-Wilk test. As they were normally distributed, the results were tested for significance by independent *t*-test and the adjusted statistical value after Bonferonni correction was *p* < 0.0018. Threshold for clinically relevant differences between the cleft and non-cleft groups was set at 3 mm^35^.

### Intra-observer and inter-observer reliability

Results for intra-observer and inter-observer reliabilities are illustrated in Table 1. Intra-class correlation for all landmarks ranged from 0.76 to 0.99 for intra-observer reliability. This indicated good to excellent reliability of all the soft tissue landmarks. In addition, the mean difference for all linear measurements was less than 1 mm, which can be deduced as high precision. Meanwhile, intra-class correlation for inter-observer reliability of all landmarks ranged from 0.54 to 0.99. This indicated fair to excellent reliability of all the soft tissue landmarks. Four variables revealed mean difference between 1 and 1.5 mm (left ocular width, pronasale to right alar base, pronasale to left alar base and left upper lateral lip length) and one variable (right ocular width) showed 1.55 mm mean difference. Nevertheless, these values still suggest moderate precision.

### Facial measurement for the orbital region

Facial measurements of normal and cleft subjects in the orbital region are detailed in Table 2. Generally, the cleft group had larger measurements compared to the normal group but the differences were insignificant (*p* > 0.0018). The intercanthal widths were greater in subjects with UCLP than in control subjects but the mean difference was not significant (*p* = 0.33) and clinically negligible (0.81 mm).

### Facial measurement for the nose region

Table 3 displays the facial measurements of normal and cleft subjects in the nose region. Horizontal dimensions of the nose showed significant discrepancies between the cleft and normal groups except in the alar base width. The cleft group demonstrated an insignificantly wider alar base width compared to the normal group with a small mean difference of 1.1 mm. However, the alar base root width was significantly broader in the cleft group with a mean difference of 3.19 mm (*p* = 0.00), which is clinically significant.

Distances of alar base root to the ear insertion (oR–sbalR and oL–sbalL) on both right and left sides are significantly narrower for the cleft group with mean differences of 5.63 mm (*p* = 0.00) and 6.86 mm (*p* = 0.00) respectively. These differences are considered clinically relevant as the values exceed the 3 mm threshold that was set. Right alar length (prn–alR) was significantly shorter in the cleft group compared to the normal group (*p* = 0.00). However, this difference was not clinically relevant as the mean difference was less than 3 mm.

Left nostril floor width (sn–sbalL) of the cleft group was wider than the normal group. This difference is statistically and clinically significant due to the mean difference of 3.83 mm (*p* = 0.00). Nevertheless, the control group had significantly wider right nostril floor width (sn–sbalR) although the mean difference is negligible and therefore clinically irrelevant (*p* = 0.00). Vertically, the cleft group had shorter nose dorsum length but this difference is not significant (*p* = 0.49).

### Facial measurement for the mouth region

Upper vermillion thickness was significantly shorter in the cleft group (*p* = 0.00). However, the value is not clinically significant as the mean difference is only 2.76 mm. In addition, the right upper lateral lip length was significantly longer in the cleft group (*p* = 0.00). Nevertheless the difference was clinically irrelevant. Mouth width was narrower in the cleft group but this difference is not significant (*p* = 0.09) (Table 4).

### Facial measurement for the face heights and other ratios

Table 5 demonstrates the face heights and other ratios of normal and cleft subjects. In general, the cleft group displayed insignificantly shorter face heights and lower face height ratio when compared to the normal group (*p* > 0.05). The mean differences in face heights were also clinically insignificant (less than 3 mm). Nose to mouth width ratio was wider in the cleft group but this was insignificant (*p* = 0.57) and clinically irrelevant as the mean difference is very small (0.04 mm).

## Discussion

3D surface imaging systems can be roughly divided into laser-based and photography-based (photogrammetry) systems. Although early attempts at stereophotogrammetry were technically cumbersome and computationally intensive, the system has been updated and restructured with the advent of newer 3D photogrammetric devices. The 3D VECTRA M5 photosystem was utilised in this study and consists of five camera pods, making it possible to produce high resolution, photorealistic images. The validation study has been reported in detail by Metzler *et al.*^32^. The system showed high precision and accuracy for the determination of landmarks and measurements. They also concluded that the five-pod 3D photosystem is suitable for clinical applications, particularly in anthropometric studies. A similar system based on stereophotogrammetry has been employed in the investigation of facial dimensions of Malay children with repaired UCLP aged 8 to 10 years old^20^. A recent anthropometric study^26^ performed on infants with unilateral cleft lip also utilised a hand-held 3D imaging system, VECTRA H-1 (Canfield Scientific Co. Ltd., Fairfield, NJ). This form of non-invasive imaging with quick capture speed would appear to be the most promising technique for overcoming the lack of cooperation likely in small children.

It has been postulated that the growth retardant effect of cleft surgical procedures can be demonstrated transversely as high as the orbital level in view of findings that UCLP and bilateral CLP subjects have narrower intercanthal widths than the controls^17^. Another study by Yamada *et al.*^36^ concluded that compared with normal Japanese children, the affected children between 4 to 18 months of age have wider intercanthal distance. However, there was no significant difference between the intercanthal distance of older children (4 years old) and normal children. In this present study, intercanthal width of UCLP patients is wider when compared to the normal subjects but the finding is not significant as then mean difference is too small. This finding concurs with research studies conducted by Yamada *et al.*^36^ and Zreaqat *et al.*^20^ which also found that although intercanthal width in children affected with UCLP tended to be narrower than their normal counterparts, the difference is insignificant. Possible explanation for the discrepancy between the present study and Duffy *et al.*^17^ might be due to the variety in research samples. Although both studies had almost similar age range, and comparable inclusion and exclusion criteria, they were conducted on two different populations. Duffy *et al.*^17^ analysed 3D images of the Caucasian child cleft face in the study which took place in London, United Kingdom. The natural differences between the Oriental and Caucasian features might have contributed to the different findings.

Othman *et al.*^25^ conducted a similar study of 3D analysis of facial morphology in Malaysia, comparing adult patients with repaired, non-syndromic, complete UCLP with a control group without clefts. They found that there was a significant difference in the intercanthal widths, whereby the intercanthal width was wider in the cleft group with a mean difference of 3.88 mm. In the paper, they have proposed 5 mm as an appropriate threshold to indicate clinical relevance. Therefore, even though the difference in intercanthal width was statistically significant, it was deemed clinically irrelevant. Ethnic Malay patients were used with older samples (18–25 years), and the quantitative values of measurements were assessed using proportion indices. Interestingly, it can be observed that UCLP adults of Malay ethnicity have a wider intercanthal dimension compared to UCLP children of Chinese ethnic background. The discrepancy might be due to the difference in the age group, growth and ethnicity.

The findings that UCLP patients have broader alar base width, wider alar base root width, flatter nose, and wider left nostril floor width are in keeping with several previous studies^17^,^20^,^36^. These concur with findings by Krimmel *et al.*^37^ who reported that the highest degree of cleft deformity was seen in the horizontal dimensions of the nose. Zreaqat *et al.*^20^ hypothesised the horizontal discrepancies could be due to the inability of primary surgeries to restore the loss of medial insertion points of the facial muscles. The cleft group demonstrated an insignificantly larger alar base width compared to the normal group with a small mean difference of 1.1 mm. As the clinically relevant mean difference in this study was set at 3 mm, the difference was considered clinically insignificant. The result of a study by Duffy *et al.*^17^, which detected an insignificantly wider alar base width with a mean difference of 1.9 mm, is in accord with our finding. Meanwhile, Zreaqat *et al.*^20^ observed a significantly wider alar base width in cleft children of Malay ethnicity with a mean difference of 2.89 mm. Several studies investigating facial morphology of adult patients with repaired UCLP using proportion indices also observed a wider alar implantation in their cleft group^3^,^25^,^38^.

Distances of alar base root to the ear insertion (oR–sbalR and oL–sbalL) on both right and left sides are significantly narrower for the cleft group. These differences are considered clinically relevant as the values exceed the 3 mm threshold that was set. Zreaqat *et al.*^20^ found a statistically significant difference of the alar base root to ear insertion distance on the left side but not on the right. However, the mean difference was too small (0.35 mm) to be considered clinically relevant. Alar base root width was significantly broader in the cleft group. This result concurs with findings of other studies^17^,^20^. However, Zreaqat *et al.*^20^ observed a mean difference of only 0.82 mm which is clinically irrelevant according to the threshold that we had set. Measurements of left alar length (prn–alL) and left nostril floor width (sn–sbalL) were significantly larger in the cleft group compared to the normal. It is observed that mean differences of left alar length and left nostril floor width were greater than those discovered in the study conducted by Zreaqat *et al.*^20^. The difference in these findings might be influenced by the difference in ethnicity albeit the similarity in age group and classification of clefting. The discrepancies observed in the cleft group gave rise to the residual deformities in the nose region commonly encountered in cleft patients regardless of the surgical repair that they had undergone. The alar is flattened and elongated giving the appearance of a wider and flatter nose. The increase in horizontal nasal dimension might be due to interruption of orbicularis oris at muscular level therefore displacing the columella towards the non-cleft side and the nasal tip to the opposite side^39^.

The effect of surgery on lip length is intimately related to the type of surgical repair with different procedures resulting in either a shorter or longer lip. A long lip was more often associated with the Z-plasty type repair group of patients, and short lips were more associated with Millard repair and straight line repair^40^. In the present study, the majority of the cleft patients received either Millard or Tennison lip repair. Their upper vermillion thickness was significantly shorter when compared to their normal counterparts. This result is in accordance with findings by Assuncao^40^. Duffy *et al.*^17^ and Zreaqat *et al.*^20^ also demonstrated shorter upper lip length with deficient upper vermilion thickness in their cleft subjects. Although statistically insignificant, the mouth width was narrower in the cleft group and this result is mirrored in the findings of Duffy *et al.*^17^ and Zreaqat *et al.*^20^. It is hypothesized that tissue deficiency, marginal excisions, scar contraction, and growth disruption are all likely to have contributed to the reduced mouth dimensions in the cleft subjects^17^. The nose/mouth width ratio has been suggested as important in the assessment of the CLP face with a ratio significantly above the normal being considered as potentially unattractive^3^. The current study demonstrated an increase in the nose/mouth width ratio in the cleft group when compared to the normal group. This is expected due to the earlier findings of wider alar implantation but reduced mouth dimension. Our finding contradicts the result displayed by Zreaqat *et al.*^20^ which stated the ratio is smaller in their cleft group.

Current study only employed qualitative analysis of linear facial measurements between specific facial landmarks. More sophisticated 3D analysis method such as surface matching or the advanced technology known as dense correspondence surface technique and generic facile mesh have been explored. Unfortunately with the current 3D camera, these techniques were unable to perform in the present study due to lack of expertise.

Within the limitations and based on the results of the present study, it can be concluded the faces of Chinese children with repaired UCLP displayed meaningful differences when compared to the normal group mainly in the nasolabial regions, where the Chinese children with repaired UCLP exhibited a wider and flatter nose, wider nostril floor width, shorter upper lip length and thinner upper vermillion. It is hoped that by providing the facial measurements of Chinese children with repaired UCLP and comparing them with their normal counterparts, the data could be used to evaluate the outcome of CLP surgery. This study also has the potential to assess the long term effects of surgery on growth in the long run. It can be suggested that the use of non-invasive 3D stereophotogrammetric imaging technology has the potential to evaluate the outcome of surgical repair on the cleft face.

## Material and Methods

### Study design

This was a comparative, cross-sectional study designed to investigate the facial dimensions of UCLP subjects and to compare the measurements with their normal counterparts. All experimental protocols were approved by Medical Ethics Committee, Faculty of Dentistry, University of Malaya, Kuala Lumpur. Ethics Committee/IRB Reference Number: DF CD 1301/0009(P). The methods were carried out in accordance with the approved guidelines.

### Sampling and sample

Recruitment of subjects took place from January 2013 to November 2014. Twenty Chinese children aged 7 to 12 years old (mean age 10.22 years; standard deviation 1.81) with repaired UCLP (15 left side cleft and 5 right side cleft) were recruited from the Combined Cleft Lip and Palate Clinic, Faculty of Dentistry, University of Malaya and also from the cleft database. Participants were required to complete a questionnaire to verify their ethnicity. The following selection criteria were used:

1. Third generation Chinese ethnicity.
2. Age between 7 and 12 years.
3. Non-syndromic UCLP (confirmed by clinical geneticist).
4. Had not undergone alveolar bone grafting, no previous orthodontic treatment including face mask treatment.

All of the affected children had primary reconstructive surgery performed by one of three surgeons following a similar surgical protocol. Cheiloplasty was performed at the age of 3 months with nasal repair performed at the same time if it was deemed necessary by the surgeons. At the age of 9 months, palatoplasty was performed. Children with mixed ethnicities, had undergone alveolar bone grafting, and those with cleft as part of craniofacial syndrome were excluded.

The control group comprised of 40 Chinese children aged 7–12 years old (mean age 10.03 years; standard deviation 1.51). They were enrolled from the Paediatric Dentistry treatment waiting list, Faculty of Dentistry, University of Malaya. Subjects that fulfilled the following inclusion criteria were recruited:

1. Third generation Chinese ethnicity.
2. Age between 7 and 12 years.
3. Class I incisor relationship according to British Standard Institute^41^.

Patients with mixed ethnicities, who had history of orthodontic treatment or who were currently on active treatment and with craniofacial deformities, were excluded from this study.

The power and sample size calculation was done using PS Software Version 3.0.43 2011^42^. In a previous study which looked into the facial dimensions of Malay children affected with UCLP, the result of alar base width within each group was normally distributed with standard deviation of 2.92^20^. Measurement of alar base width was selected as Krimmel *et al.*^37^ reported that the highest degree of cleft deformity was seen in the horizontal dimensions of the nose. In addition to that, alar base width was one of the most significantly different measurements from that found in controls. A possible difference of 3 mm between groups was selected based on a study, which reported differences of up to 2 mm between the two hemifaces are considered to fall within a normal range^35^. Therefore, with a sample size of 18 in each group, there will be 85% power to detect at least 3 mm difference in facial measurement with alpha at 0.05. Taking into consideration the possibility of dropouts, 20 samples were required for UCPL group and 40 for the control group. All suitable subjects were given verbal and written explanation and invited to join the research. Written consent was obtained from the parents or legal guardians of the children who agreed to participate.

### Image capture, measurement, and analysis of 3D images

A 3D photograph of all suitable subjects was captured using the VECTRA-M5 360 (Canfield Scientific Inc, Fairfield, NJ, USA) camera available in the 3D lab, Dental Faculty of University of Malaya for full face imaging. The VECTRA-M5 360 camera consisted of 3D stereophotograms. It consisted of a five-pod system with a pair of lens for every pod. These five pairs of identical cameras were separated by a known base distance. The 3D model image constructed from the fusion of ten images acquired by these ten lenses will give a 360 degree imaging of the subject to ensure image consistency and magnification. Prior to every imaging session, VECTRA M5-360 Head System calibration was performed according to the manufacturer’s instructions. Calibration of the Vectra 3D system ensures that the relationship of the cameras and other components is understood and recorded by the system. This is critical to creating geometrically accurate 3D models. Thus, calibration is performed daily, prior to photo taking and whenever the system has been moved. A calibration target comprising discs on contrasting background and of accurately known dimensions and location is presented and captured by the cameras for a variety of target poses. Images of the target from all the cameras are processed to find the central location of the discs and these coordinates are used to fit an approximate geometric model of each camera and its respective relative orientation to the target^43^.

Prior to capture, the subject was instructed to wear a tightly fitted head cap to ensure that hair was secured away from the face and ears. The subject was then seated in front of the camera on a self-adjustable stool, and the operator monitored the position of the subject’s head so that multiple images can be seen in the live video preview. Facial expression must be in repose or neutral. When subject is correctly positioned, images of the face were captured. Colour texture images will be displayed on the computer screen, immediately followed by monochrome surface images. Green and red elements will begin to appear on the images for the system to build the 3D model. The Vectra 3D photosystem captured several carefully synchronised 2D digital camera views of the subject and then used photogrammetric algorithms to compute a highly accurate map of 3D shape and colour coordinates of the observed surface. Capture process occurred in less than 2 milliseconds. The automatic 3D processing took less than a minute; with the resulting 3D image being stored as a file on the computer and displayed on the screen.

**Figure 1:**
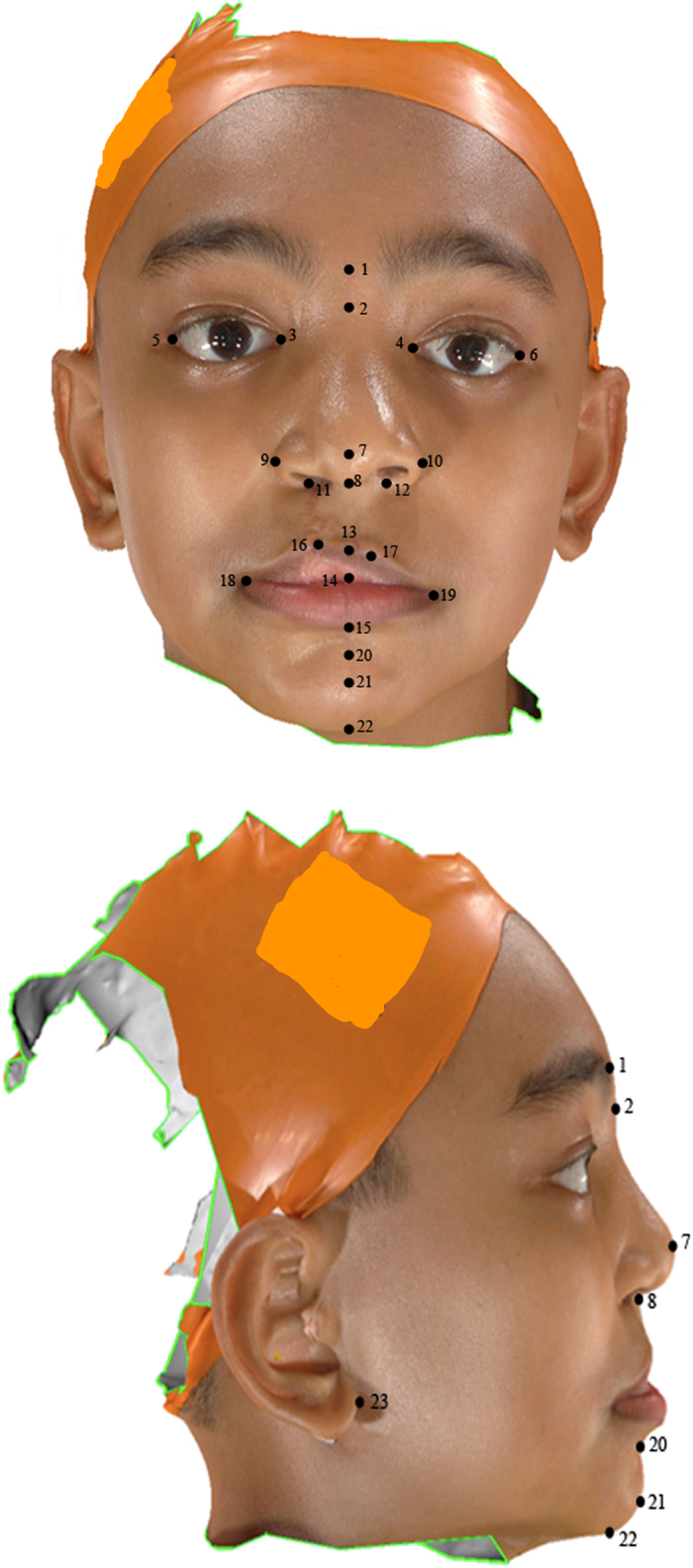
The distribution of soft tissue landmarks. Frontal and lateral views.

Using Mirror software (Canfield, Fairfield, NJ), all 5 right unilateral sided clefts were reflected to become left unilateral sided clefts. This was to ensure the uniformity of analysis and to reduce biases. Thirty anthropometric landmarks employed in this study were proposed and defined by Farkas^44^ (Table 6). The distribution of landmarks was illustrated in Fig. 1. A set of 25 linear distances and 2 ratios was calculated from the landmark coordinates (Table 7). These distances correspond to traditional facial anthropometric measurements as defined by Farkas *et al.*^38^, Duffy *et al.*^17^, and Zreaqat *et al.*^20^, and were chosen for their clinical relevance and because they comprised a core set of variables for describing facial analysis of a cleft patient. Landmarks on the 3D facial images were digitised manually using Mirror software.

### Method error

As landmark identification improves with training and operator experience, training and calibration with an expert individual is necessary prior to commencement of the main study. A research assistant with six years’ experience in 3D imaging was employed to train the operator with landmark placement. 3D images of 10 subjects (5 cleft subjects and 5 normal subjects) were randomly chosen from the University of Malaya 3D database. After a series of training regimens, 24 anthropometric landmarks were manually digitised on each image by both the operator and an experienced research assistant. Landmarks were digitised with a two-week interval between measurements to prevent memory-biased placement of landmarks. A set of 25 linear distances was then calculated from the landmark coordinates. Intra-class correlation coefficient test (ICC) was used to determine the intra-observer and inter-observer reliability. To further support the reliability of repeated measurements, a Wilcoxon Rank test was performed to detect any significance of mean difference for each inter-landmark distance, with *p* value less than 0.05 being considered significant. Fleiss^45^ and Roberts and Richmond^46^ suggested that ICC value below 0.4 constitutes poor reliability, between 0.4 and 0.75 signifies fair to good reliability, whilst above 0.75 represents excellent reliability. The tolerance thresholds for mean difference used by Aldridge *et al.*^47^ and Mutsvangwa *et al.*^48^ were used in this study to classify precision for the inter-landmark distances. A difference of less than 1 mm between two sets of measurements was considered highly precise; between 1 and 1.5 mm, precise; from 1.6 to 2 mm, moderately precise; and anything more than 2 mm, imprecise.

### Statistical analysis

Pearson Chi-Square test was performed to test if there was any sexual differences at baseline. Basic statistical analysis, normality of data distribution and independent *t*-test to detect the significant differences between the cleft and normal groups was carried out using SPSS Version 12.0.1 (SPSS for Windows, SPSS Inc., Chicago, IL, USA). Bonferroni adjustment was also performed to avoid Type 1 error. The initial level of significance was set at *p* < 0.05 and the adjusted statistical value after Bonferonni correction was p < 0.0018. For clinically significant differences, a threshold of 3 mm was selected based on a study, which reported that differences of up to 2 mm between the two hemifaces are considered to fall within the normal range^35^.

## Additional Information

**How to cite this article**: Othman, S. A. and Koay, N. A. A. Three-dimensional facial analysis of Chinese children with repaired unilateral cleft lip and palate. *Sci. Rep.* **6**, 31335; doi: 10.1038/srep31335 (2016).

## Figures and Tables

**Table 1:** ICC and mean differences of intra and inter-observer reliabilities.

Variables	Intra-observer reliability		Inter-observer reliability	
	ICC	Mean difference (mm)	ICC	Mean difference (mm)
Biocular width	0.98	0.79	0.91	0.97
Right ocular width	0.93	0.46	0.69	1.55
Left ocular width	0.90	0.39	0.76	1.15
Intercanthal width	0.99	0.04	0.99	0.09
Right endocanthion to nasion	0.92	0.22	0.89	0.07
Left endocanthion to nasion	0.93	0.46	0.86	0.15
Right otobasioninferius to right subalare	0.99	0.03	0.72	0.15
Left otobasioninferius to left subalare	0.99	0.47	0.79	0.36
Alar base width	0.99	0.42	0.81	0.60
Pronasale to right alar base	0.76	0.52	0.54	1.18
Pronasale to left alar base	0.94	0.54	0.63	1.06
Alar base root width	0.96	0.35	0.74	0.98
Subnasale to right alar base root	0.98	0.30	0.82	0.83
Subnasale to left alar base root	0.99	0.08	0.90	0.62
Nose dorsum length	0.97	0.06	0.57	0.41
Mouth width	0.98	0.50	0.98	0.05
Upper lip length	0.91	0.07	0.86	0.33
Lower lip length	0.96	0.55	0.62	0.72
Upper vermillion thickness	0.99	0.12	0.98	0.36
Lower vermillion thickness	0.98	0.24	0.92	0.46
Right upper lateral lip length	0.93	0.02	0.68	0.95
Left upper lateral lip length	0.94	0.37	0.57	1.13
Lower face height	0.98	0.25	0.99	0.10
Upper face height	0.99	0.17	0.84	0.41
Total face height	0.99	0.37	0.88	0.27

**Table 2:** Facial measurements of normal and cleft subjects in the orbital region.

Measurement	UCLP group (n = 20) Mean (SD)	Control group (n = 40) Mean (SD)	Mean difference (mm) (95% CI)	*t* statistic d (*f*)	*p* value
Biocular width	88.71 (3.87)	87.77 (4.86)	−0.94 (−3.44, 1.55)	−0.76 (58)	0.45
Right ocular width	27.63 (1.84)	27.42 (1.79)	−0.21 (−1.20, 0.79)	−0.41 (58)	0.68
Left ocular width	27.70 (1.88)	27.38 (1.70)	−0.32 (−1.28, 0.64)	−0.66 (58)	0.51
Intercanthal width	36.01 (2.07)	35.20 (3.42)	−0.81 (−2.49, 0.86)	−0.98 (58)	0.33
Right endocanthion to nasion	21.46 (1.10)	20.93 (2.06)	−0.53 (−1.52, 0.46)	−1.07 (58)	0.29
Left endocanthion to nasion	21.44 (1.68)	21.43 (1.95)	0.02 (−1.04, 1.01)	−0.03 (58)	0.90

**Table 3:** Facial measurements of normal and cleft subjects in the nose region.

Measurement	UCLP group (n = 20) Mean (SD)	Control group (n = 40) Mean (SD)	Mean difference (mm) (95% CI)	*t* statistic d (*f*)	*p* value
Right otobasioninferius to right subalare	90.91 (4.58)	96.54 (6.67)	5.63 (2.31, 8.96)	3.40 (58)	0.00*
Left otobasioninferius to left subalare	88.82 (3.27)	95.69 (6.84)	6.86 (4.25, 9.48)	5.26 (58)	0.00*
Alar base width	36.37 (1.95)	35.27 (2.71)	−1.10 (−2.47, 0.26)	−1.61 (58)	0.11
Pronasale to right alar base	20.44 (2.27)	23.04 (2.54)	2.59 (1.25, 3.94)	3.86 (58)	0.00*
Pronasale to left alar base	25.01 (1.88)	23.67 (2.60)	−1.32 (−2.63, −0.01)	−2.02 (58)	0.04
Alar base root width	26.28 (1.84)	23.08 (1.76)	−3.19 (−4.17, −2.21)	−6.53 (58)	0.00*
Subnasale to right alar base root	10.74 (1.39)	11.89 (1.08)	1.15 (0.49, 1.80)	3.53 (58)	0.00*
Subnasale to left alar base root	16.71 (1.23)	12.88 (1.07)	−3.83 (−4.44, −3.21)	−12.40 (58)	0.00*
Nose dorsum length	35.04 (4.67)	36.12 (6.15)	1.09 (−2.04, 4.21)	0.69 (58)	0.49

^*^Statistical significance value after Bonferroni adjustment: *p* < 0.0018.

**Table 4:** Facial measurements of normal and cleft subjects in the mouth region.

Measurement	UCLP group (n = 20) Mean (SD)	Control group (n = 40) Mean (SD)	Mean difference (mm) (95% CI)	*t* statistic d (*f*)	*p* value
Mouth width	39.20 (3.25)	41.57 (5.88)	2.37 (−0.45, 5.21)	1.68 (58)	0.09
Upper lip length	19.08 (2.24)	20.87 (3.24)	1.79 (−0.17, 3.41)	2.21 (58)	0.03
Lower lip length	17.14 (1.12)	17.03 (3.97)	−0.11 (−1.93, 1.71)	−0.12 (58)	0.91
Upper vermillion thickness	6.60 (1.75)	9.34 (1.94)	2.76 (1.74, 3.79)	5.38 (58)	0.00*
Lower vermillion thickness	8.86 (1.44)	9.05 (1.93)	0.19 (−0.78, 1.18)	0.41 (58)	0.69
Upper right lateral lip length	15.15 (1.67)	13.33 (1.95)	−1.82 (−2.84, −0.79)	−3.55 (58)	0.00*
Upper left lateral lip length	15.24 (2.17)	13.98 (1.85)	−1.25 (−2.32, −0.17)	−2.32 (58)	0.02

^*^Statistical significance value after Bonferroni adjustment: *p* < 0.0018.

**Table 5:** Face heights and other ratios of normal and cleft subjects.

Measurement	UCLP group (n = 20) Mean (SD)	Control group (n = 40) Mean (SD)	Mean difference (mm) (95% CI)	*t* statistic d (*f*)	*p* value
Lower face height	60.62 (6.07)	62.76 (4.62)	2.14 (−0.68, 4.96)	1.52 (58)	0.13
Upper face height	46.41 (4.48)	47.27 (4.17)	0.85 (−1.48, 3.19)	0.73 (58)	0.47
Total face height	105.55 (8.99)	107.57 (6.12)	2.02 (−2.54, 6.59)	0.91 (58)	0.91
LFH/TFH percentage	57.41 (2.57)	58.35 (2.85)	0.94 (−0.58, 2.45)	−1.24 (58)	0.22
Nose/mouth width ratio	0.93 (0.08)	0.88 (0.34)	−0.04 (−0.19, 0.11)	−0.57 (58)	0.57

**Table 6:** Anthropometric landmarks.

No	Landmark	Definition
1.	Glabella (gla)	Midline point between eyebrows
2.	Soft tissue nasion (N)	Deepest concavity point on frontonasion suture
3.	Endocanthion right (enR)	Right inner commisure point of eye fissure
4.	Endocanthion left (enL)	Left inner commisure point of eye fissure
5.	Exocanthion right (exR)	Right outer commisure point of eye fissure
6.	Exocanthion left (exL)	Left outer commisure point of eye fissure
7.	Pronasale (prn)	Most prominent midline point on the nose tip
8.	Subnasale (sn)	Midline junction point between columella and upper lip
9.	Alare right (alR)	Most lateral point on the alar contour on the right
10.	Alare left (alL)	Most lateral point on the alar contour on the left
11.	Subalare right (sbalR)	Right point on the lower margin of nasal alar base where alar disappears into the upper lip skin
12.	Subalare left (sbalL)	Left point on the lower margin of nasal alar base where alar disappears into the upper lip skin
13.	Labralesuperius (ls)	Midpoint of upper vermillion line
14.	Stomion (sto)	Midpoint between upper and lower lip
15.	Labraleinferius (li)	Midpoint of lower vermillion line
16.	Crista philtri right (cphR)	Junction between upper lip vermillion and philtral peak on the right
17.	Crista philtri left (cphL)	Junction between upper lip vermillion and philtral peak on the left
18.	Cheilion right (chR)	Right labial commisure point
19.	Cheilion left (chL)	Left labial commisure point
20.	Soft tissue B point (b)	Deepest concavity on the anterior profile of mandible
21.	Soft tissue pogonion (pog)	Most anterior midpoint of chin
22.	Soft tissue gnathion (gn)	Most inferior midpoint of chin
23.	Otobasioninferius right (oR)	Inferior insertion of the ear on the right
24.	Otobasioninferius left (oL)	Inferior insertion of the ear on the left

**Table 7:** Linear distances.

No	Variables	Anthropometric notation
1.	Biocular width	ex–ex
2.	Right ocular width	exR–enR
3.	Left ocular width	exL–enL
4.	Intercanthal width	enR–enL
5.	Right endocanthion to nasion	enR–n
6.	Left endocanthion to nasion	enL–n
7.	Right otobasioninferius to right subalare	oR–sbalR
8.	Left otobasioninferius to left subalare	oL–sbalL
9.	Alar base width	alR–alL
10.	Pronasale to right alar base	prn–alR
11.	Pronasale to left alar base	prn–alL
12.	Alar base root width	sbalR–sbalL
13.	Subnasale to right alar base root	sn–sbalR
14.	Subnasale to left alar base root	sn–sbalL
15.	Nose dorsum length	n–prn
16.	Mouth width	ch–ch
17.	Upper lip length	sn–sto
18.	Lower lip length	sto–b
19.	Upper vermillion thickness	ls–sto
20.	Lower vermillion thickness	li–sto
21.	Right upper lateral lip length	sbalR–cphR
22.	Left upper lateral lip length	sbalL–cphL
23.	Lower face height (LFH)	sn–gn
24.	Upper face height	n–sn
25.	Total face height (TFH)	n–gn
26.	LFH/TFH percentage	(sn–gn/n–gn) x 100%
27.	Nose base/mouth width ratio	al–al/ch–ch
